# Cytokine responses in primary chicken embryo intestinal cells infected with *Campylobacter jejuni *strains of human and chicken origin and the expression of bacterial virulence-associated genes

**DOI:** 10.1186/1471-2180-8-107

**Published:** 2008-06-27

**Authors:** Yi-Ping Li, Hanne Ingmer, Mogens Madsen, Dang D Bang

**Affiliations:** 1 Department of Poultry, Fish and Fur Animals, National Veterinary Institute (VET), Technical University of Denmark (DTU), Hangøvej 2, DK-8200 Aarhus N, Denmark; 2 Dianova, Technical University of Denmark (DTU), Hangøvej 2, DK-8200 Aarhus N, Denmark; 3 Department of Veterinary Pathobiology, Life Science Faculty, University of Copenhagen, Stigboejlen 4, DK-1870 Frederiksberg C, Denmark

## Abstract

**Background:**

*Campylobacter jejuni *is a major cause of inflammatory diarrhoea in humans and is considered a commensal of the gastroenteric tract of the avian host. However, little is known about the interaction between *C. jejuni *and the avian host including the cytokine responses and the expression of the bacterial genes. We have investigated the invasiveness of primary chicken embryo intestinal cells (CEICs) by *C. jejuni *strains of human and chicken origins and the production of pro-inflammatory cytokines as well as the expression of the bacterial virulence-associated genes during co-cultivation.

**Results:**

*C. jejuni *strains are capable of invading the CEICs and stimulate these cells in a pro-inflammatory manner and during this interaction the expression of the bacterial virulence-associated genes *ciaB, dnaJ *and *racR *is increased. Furthermore, incubation of bacteria with conditioned cell- and bacteria-free media from another co-cultivation experiment also increased the expression of the virulence-associated genes in the *C. jejuni *chicken isolate, indicating that the expression of bacterial genes is regulated by component(s) secreted upon co-cultivation of bacteria and CEICs.

**Conclusion:**

We show that under *in vitro *culture condition *C. jejuni *strains of both human and chicken origins can invade avian host cells with a pro-inflammatory response and that the virulence-associated genes of *C. jejuni *may play a role in this process.

## Background

*Campylobacter *is a spiral Gram-negative, thermophilic, obligate microaerobic bacterial genus that is ubiquitous in temperate environments [1]. *Campylobacter jejuni *is recognized as the leading cause of bacterial food-borne and water-borne enteric diarrhea in humans. In the United States and Great Britain, more than 1% of the population is infected with *C. jejuni *each year [2,3], and the incidence of *C. jejuni*-induced disease may be even higher in developing countries, where infection often goes unreported. The clinical symptoms of *Campylobacter *infection include watery to bloody diarrhea, abdominal pain, fever, headache, nausea and vomiting in acute infection, even a severe inflammation of the intestinal mucosa with an influx of professional phagocytes [4]. Mostly the infection of *Campylobacter *is self-limiting, but some infections are associated with more serious medical sequels such as Reiter's syndrome (reactive arthritis), Miller-Fisher syndrome (MFS) and Guillain-Barré syndrome (GBS) [5]. The *Campylobacter*-associated illness has become an economic and health burden in the world [3,6]. Although knowledge about the organism and host responses to infection has been growing rapidly in the past decades, the pathogenesis of *C. jejuni *is not yet well understood.

*C. jejuni *colonizes the intestinal mucosa of warm-blooded hosts, including farm animals and humans. Among these hosts the favored environment appears to be the intestines of avians, including chickens, that provide optimal temperature conditions for growth, e.g. 42°C. Unlike in the colonization of humans, *C. jejuni *colonizes the intestinal tracts of most mammals and birds at a high level with little or no pathology [7]. The differences of pathology between human and chicken may relate partly to the host immune system. Cytokines are central to the development of effective immunity against microbial pathogens. Previous studies have shown that *C. jejuni *can invade human-derived epithelial cell cultures [8,9] and induce the production of a number of cytokines and chemokines during the course of experimental infections [10-15], as well as in clinical *Campylobacter *infections [16]. In general, cytokine responses have mainly been monitored on human-derived cell lines with only a few studies performed on chicken cells [15,17] and none involving *C. jejuni *isolates of chicken origin. Furthermore, experimental colonization experiments have shown that passage through the chicken gut enhances the colonization potential and virulence of *C. jejuni *[18], suggesting that it is important to use *C. jejuni *of chicken origin in order to more closely mimic the interactions taking place in the avian gut. Therefore, we used primary chicken embryo intestinal cells (CEICs) as a model to compare the cytokine responses of CEICs elicited by *C. jejuni *strains of human and chicken origin, as well as the concomitant expression of the virulence-associated genes of *C. jejuni*.

## Results

### Intracellular bacterial counts

**Figure 1:**
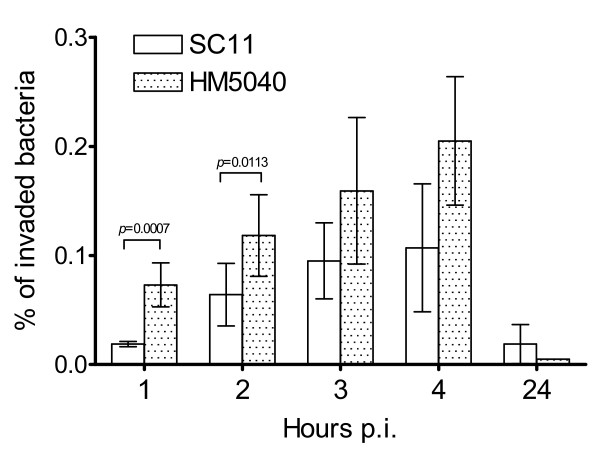
**Invasion of primary chicken embryo intestinal cells (CEICs) by *C. jejuni***. Cells grown at equivalent levels (1.7 × 10^5 ^cells/well) were inoculated at an MOI of 100:1 with either *C. jejuni *strain SC11 (chicken isolate) or HM5040 (human isolate). The number of internalized bacteria was assessed by the gentamycin protection assay at 1, 2, 3, 4 and 24 h post inoculation (p.i.). Data are representative of three independent experiments (average ± SD).

With the aim of determining whether *C. jejuni *is able to invade primary chicken embryo intestinal cells (CEICs), intracellular bacterial counts were performed using the gentamicin protection method [8]. For this purpose two *C. jejuni *strains, a Danish chicken isolate SC11 and a clinical human isolate HM5040, were investigated. Prior to the infection experiment, the motility and morphology of both isolates was checked under light microscopy. The results of invasion are shown in Figure 1. The number of intracellular bacteria for both SC11 and HM5040 were gradually increased from 1 to 4 h p.i. and declined to a lower level at 24 h p.i. The percentage of internalized SC11 overall was lower than that of HM5040 from 1 to 4 h p.i., with the exception of the 24 h p.i. where the number of internalized SC11 was higher than that of HM5040. A statistically significant difference between SC11 and HM5040 was observed only at 1 and 2 h p.i. (*p *≤ 0.011).

### Production of cytokines by CEICs

**Figure 2:**
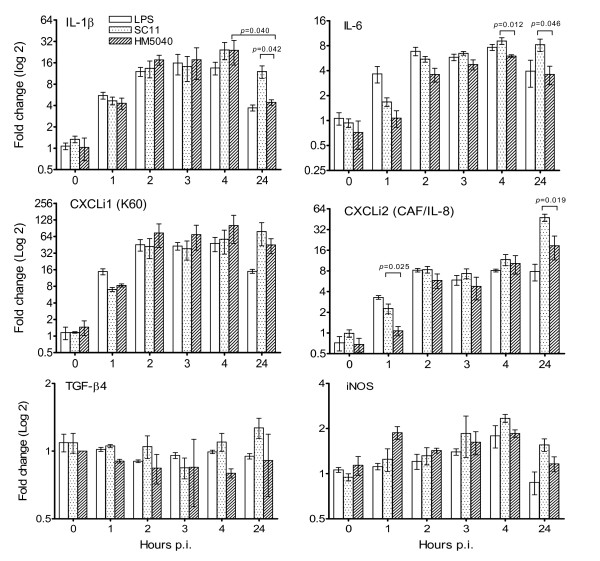
**Quantification of cytokine and chemokine transcripts from primary chicken ebmryo intestinal cells (CEICs)**. CEICs were inoculated with *C. jejuni *strain SC11 (chicken isolate) and HM5040 (human isolate) at a MOI of 100:1, RNA were isolated at various hours post inoculation (p.i.). LPS *E. coli *055:B5 (5 μg/ml) and mock inoculation were used as controls. Data are representative of three independent experiments and present as relative to mock inoculations (average ± SD) (see Methods for details).

When human cells are in contact with *C. jejuni *a strong cytokine response is induced [10,11,14]. In order to determine if a similar response can be monitored with chicken embryo intestinal cells (CEICs), the levels of IL-1β, IL-6, CXCLi1 (K60), CXCLi2 (CAF/IL-8) [19] and TGF-β4 transcripts were measured in CEICs at 0, 1, 2, 3, 4 and 24 h p.i. with *C. jejuni *strains SC11 and HM5040. Mock-inoculated cells and those inoculated with LPS (5 μg/ml) were used as controls throughout. Figure 2 shows that both bacterial strains induced the production of IL-1β, IL-6, CXCLi1 and CXCLi2, but not of TGF-β4. The level of IL-1β, IL-6 and CXCLi1 transcripts increased gradually from 1 to 4 h p.i. and declined slightly at 24 h p.i., whereas CXCLi2 transcript increased steadily throughout the observation period and reached a high level at 24 h p.i. Significant differences induced by SC11 and HM5040 were observed only at some particular time points: IL-1β at 24 h p.i. (*p *= 0.042), IL-6 at 4 p.i. (*p *= 0.012) and 24 h p.i. (*p *= 0.046) and CXCLi2 at 1 h p.i. (*p *= 0.025) and 24 h p.i. (*p *= 0.019) while IL-2 and IL-4 transcripts were undetectable. The transcript of IFN-γ was detected only at some particular time points (data not shown).

### Induction of iNOS and production of NO by CEICs

**Figure 3:**
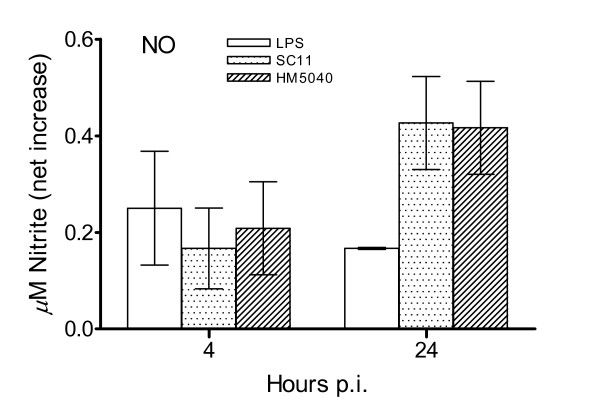
**Production of nitric oxide (NO) by primary chicken embryo intestinal cells (CEICs)**. CEICs were inoculated with *C. jejuni *strain SC11 (chicken isolate) and HM5040 (human isolate) at an MOI of 100:1, and NO in supernatants were measured at 4 and 24 hours post inoculation (p.i.). LPS *E. coli *055:B5 (5 μg/ml) and mock inoculation were used as controls. Data are representative of three independent experiments and are presented as net increases of NO, from which the NO of the mock inoculations has been subtracted (average ± SD).

To study whether there are inflammatory responses in CEICs, the cellular iNOS transcripts and the presence of NO product in the medium were measured. The level of iNOS transcripts was altered by both strains and increased gradually from 1 to 4 h p.i., followed by a decline at 24 h p.i (Figure 2). The production of NO from the inoculated CEICs was determined at 4 and 24 h p.i. using the Griess assay [20]. The results were presented as a net nitrite production over the background of the mock-inoculated CEICs (Figure 3). Nitric oxide was produced by inoculated CEICs, while as expected, the CEICs treated with LPS also showed NO induction.

### Expression of the virulence-associated genes of *C. jejuni*

**Figure 4:**
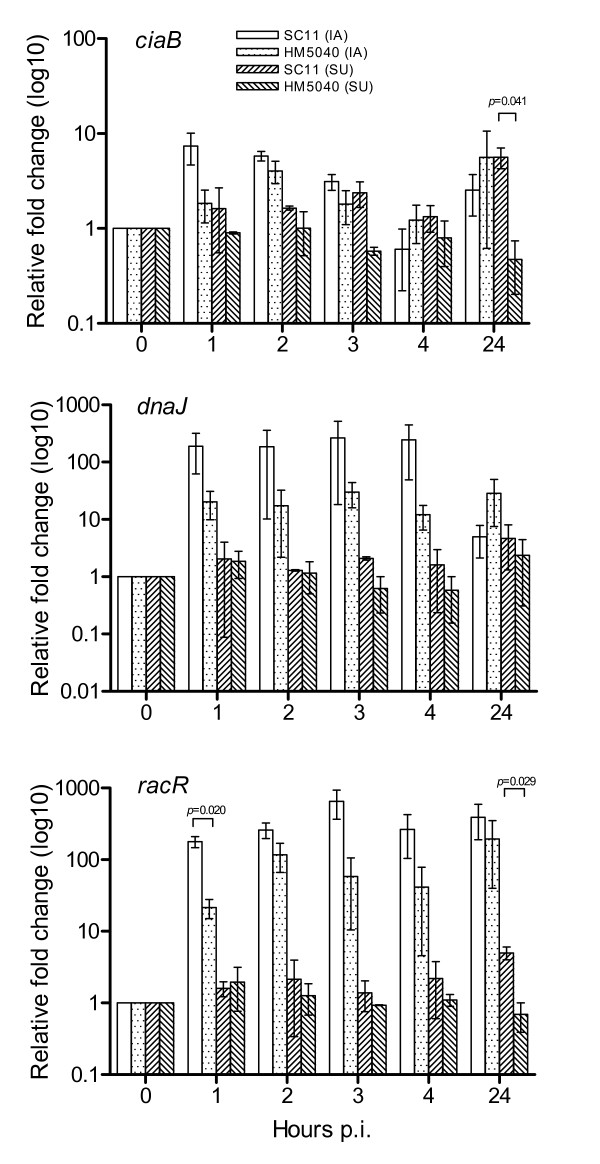
**Quantification of the expression of the virulence-associated genes of *C. jejuni***. Primary chicken embryo intestinal cells (CEICs) were inoculated with *C. jejuni *chicken strain SC11 and human strain HM5040 at a MOI of 100:1, total RNA was isolated, and the expression of *ciaB*, *dnaJ *and *racR *of *C. jejuni *in internalized/adhered (IA) and suspended (SU) bacteria were measured at various time points post inoculation (p.i.) (see Methods). Mock inoculation was used as control. Data are representative of those from three independent experiments relative to mock inoculations (average ± SD).

To study whether the contact between *C. jejuni *and CEICs induce bacterial virulence-associated genes, the expression of bacterial virulence-associated genes *ciaB*, *dnaJ *and *racR *was quantitatively determined during co-cultivation with CEICs, relative to 16S rRNA, and the fold change of the gene expression was determined relative to the mock-incubated bacteria in the absence of CEICs. The results are shown in Figure 4. The transcription of the virulence-associcated genes was increased following co-cultivation with CEICs, and the amount of present in the invaded/adhered bacteria was generally higher than that present in the suspended bacteria, especially the transcripts of *dnaJ *and *racR*. *ciaB *transcription was increased at 1 and 2 h p.i. in SC11 and HM5040, respectively, and declined to a background level at 4 h p.i., followed by a slight increase at 24 h p.i. In the suspended bacteria, the expression of *ciaB *was changed slightly, increased in SC11 and decreased in HM5040. A significant difference between SC11 and HM5040 was observed at 24 h p.i., where SC11 had a higher fold change than HM5040 had (*p *= 0.041). The transcripts of *dnaJ *and *racR *in invaded/adhered bacteria were increased in both strains throughout the course of the experiment. A statistically significant difference was observed only for *racR *at 1 h p.i. (*p *= 0.020), the level of *racR *and *dnaJ *in SC11 was higher than that in HM5040 with the exception of *dnaJ *at 24 h p.i. In suspended bacteria, the expression of *dnaJ *and *racR *was altered slightly during the inoculation. The *racR *transcript in SC11 was significantly higher than in HM5040 at 24 h p.i. (*p *= 0.029).

**Figure 5:**
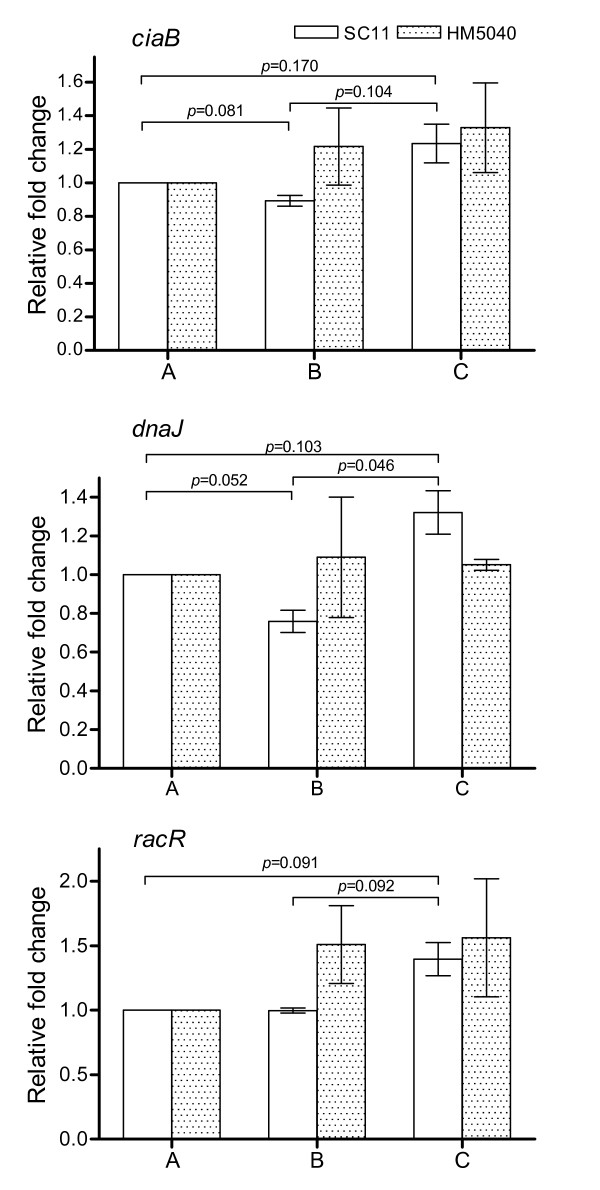
**Quantification of the expression of virulence-associated genes of *C. jejuni *under different conditions**. *ciaB*, *dnaJ *and *racR *of *C. jejuni *strains SC11 and HM5040 were measured in the bacteria incubated under different conditions, A) mock control, B) primary chicken embryo intestinal cells (CEICs) cultured medium, and C) bacteria- and cell-free supernatant from another co-cultivation experiment (see Methods). At 4 h post incubation (p.i.), total bacterial RNA was isolated, and the genes were quantitatively determined by real time quantitative PCR, relative to 16S rRNA. The level of the transcripts of mock control bacteria (A) was defined as 1, to which the level of the genes in other bacteria (B and C) was relative. Data are representative of those from three independent experiments (average ± SD).

To examine whether the induction of the virulence-associated genes is contact dependent, the expression of the selected virulence-associated genes was also measured in the bacteria that had only been incubated with bacteria- and cell-free supernatant prepared from another co-cultivation experiments. The bacteria incubated with the medium from non-inoculated CEICs and mock-incubated bacteria were used as controls. The level of expression of the virulence-associated genes in mock-incubated bacteria was defined as 1, to which the expression of the genes in the bacteria incubated with bacteria- and cell-free supernatant and cultured medium was calculated (Figure 5). After 4 hours of incubation, expression of the three virulence-associated genes *ciaB*, *dnaJ *and *racR *of SC11 was increased slightly as compared to the mock-incubated bacteria and those bacteria incubated with the medium of non-inoculated CEICs (*p *≤ 0.170). The medium of non-inoculated CEICs decreased the expression of *ciaB *and *dnaJ *in SC11 (*p *≤ 0.081), but did not alter the level of *racR *transcripts. For HM5040, expression of the three genes showed a slight increase in both bacteria incubated with the medium of non-inoculated CEICs and bacteria- and cell-free supernatant (*p *≤ 0.30).

## Discussion

In this study, primary chicken embryo intestinal cells (CEICs) were used to study the commensal interaction between chicken intestinal cells and *C. jejuni *of human and chicken origin. The results clearly show that both *C. jejuni *strains of chicken (SC11) and clinical (HM5040) origin can invade CEICs to a level approximate to that seen with human-derived cell lines [21]. However, HM5040 demonstrated a higher percentage of internalized bacteria than SC11 at early stage after inoculation (1 and 2 h p.i., Figure 1). The internalized bacteria did not persist, and declined to a low level at 24 h p.i.. These results are consistent with previous studies, in which only a few viable bacteria remained intracellularly in human dendritic cells [22] and human peripheral monocytes/macrophages [23] after a prolonged infection period (24 or 48 h) with *C. jejuni*. In contrast, other studies have demonstrated that *C. jejuni *may survive intracellular for relatively long periods of time in both phagocytes and intestinal epithelial cells [24,25], and the phagocytosis may even promote the survival of *C. jejuni *[24], which has lead to its suggested classification as a facultative intracellular pathogen. The intracellular survival may enhance the ability of *C. jejuni *to evade the host immune system, possibly favoring its long-term persistent infections [26]. However, to survive, the intracellular bacteria also have to overcome certain challenges, such as hydrogen peroxide [26] and ferrous iron [27]. For *C. jejuni *and some other enteric pathogens, it is however still largely unclear as to which primary factor(s) determines intracellular bacterial survival.

We further studied the interaction of *C. jejuni*-avian host in the aspect of the cytokine responses to *C. jejuni*. The results showed that both *C. jejuni *strains investigated were capable of inducing the expression of the pro-inflammatory cytokines IL-1β and IL-6 and the pro-inflammatory chemokines CXCLi1 and CXCLi2 in CEICs. Furthermore, we measured the production of iNOS and NO from CEICs. Inducible NOS was increased transcriptionally in CEICs at 4 h p.i. (Figure 2), and an increased NO level was also detected in the co-cultivated medium at 4 and 24 h p.i. (Figure 3). Inducible NOS, IL-1β, IL-6, and CXCLi2 are major markers of inflammatory disease. Thus, it appears that *C. jejuni *can stimulate inflammatory responses in CEICs. Our results are consistent with previous reports using avian cells, in which *Campylobacter *induced pro-inflammatory cytokines and chemokines in avian primary chick kidney cells and the avian macrophage cell line HD11 [15], as well as in primary intestinal chick cells [17]. Interleukin-8 is a pro-inflammatory cytokine, a potent chemotactic factor for many immune effector cells, and a mediator of localized inflammatory responses [28]. *Campylobacter *strains have exhibited variability in the induction of CXCL8, and those strains with a high ability to invade intestinal epithelial cells frequently induce the high levels of CXCL8 [12]. Although HM5040 showed higher invasion efficiency than SC11 shortly after inoculation, we did not observe any convincing correlation linking the invasion efficiency to the induction of cytokines and chemokines. In addition, a previous study has demonstrated that the induction of CXCL8 from INT407 cells may be regulated by two independent mechanisms, one of which requires adherence and/or invasion and the other requires cytolethal distending toxin (CDT) [29]. The functional role of the avian CXCLi2 measured in this study awaits further investigations.

Potential virulence properties of *Campylobacter *include motility, chemotaxis, colonization ability, adhesion to intestinal cells, invasion and epithelial translocation, intracellular survival, and formation of toxins and a number of putative virulence genes have been reported among the *Campylobacter *isolates [30]. *CiaB *gene and *racR *gene are highly prevalent in *Campylobacter *isolates from various sources [31], and conserved across the species [32]. *DnaJ *is a functional homologue of the *dnaJ *gene from *E. coli *and plays a role in *C. jejuni *thermotolerance and colonization of chickens [33,34]. The RacR-RacS (reduced ability to colonize) system is a two-component regulatory system and is involved in the ability to colonize the chicken intestinal tract [35]. It is important to study how these genes react to the co-cultivation as they could provide more information about *Campylobacter*-host interaction and the pathogenesis of *Campylobacter*. We thus measured the expression of these virulence-associated genes at the transcriptional level. All the three virulence-associated genes were up-regulated in invaded/adhered bacteria. The level of the expression in the suspended bacteria in contact with CEICs was generally lower than that in the internalized/adhered bacteria, which is likely a consequence of less direct contact than experienced by the internalized/adhered bacteria. Expression of *ciaB *gene of the internalized/adhered bacteria was induced to a lower extent as compared to that of *dnaJ *and *racR *(Figure 4). Previously, in *in vitro *studies have shown that *ciaB *is involved in the internalization of *C. jejuni *into host cells and required for the protein secretion process [36]. The up-regulation of the virulence-associated genes during the co-cultivation with and/or invasion into CEICs may suggest that these genes play a role in the invasion process of *C. jejuni*. Although few statistical differences were observed, SC11 showed in general a higher level of expression of these virulence-associated genes than observed in HM5040 except with a few differences (Figure 4). Shortly after inoculation (1 and 2 h p.i., Figure 1), the invasion efficiency of SC11 was significantly lower than that of HM5040. It is likely that the virulence-associated genes in SC11 are more inducible by co-cultivation with CEICs and this induction may decrease the invasion efficiency of SC11.

To study whether the induction of the virulence-associated genes is contact dependent, the bacteria were incubated with the bacteria- and cell-free supernatant from another co-cultivation experiments and with the medium of non-inoculated CEICs. The bacteria-and cell-free supernatant induced the expression of the genes tested to a greater extent in SC11 (*p *≤ 0.170) than in HM5040 (*p *≥ 0.30). The medium of non-inoculated CEICs suppressed the expression of the virulence-associated genes in chicken strain SC11, especially for genes *ciaB *and *dnaJ *(*p *≤ 0.081), while slightly increased in human strain HM5040 (Figure 5). Previous studies have showed that both host cell components and a cell-free serum-supplemented tissue culture medium can induce Cia proteins secretion, and the secretion of *C. jejuni *Cia proteins is contact dependent [37,38]. In our study, the results from SC11 may indicate that a physically contact of bacteria and CEICs is not necessary for the transcriptional up-regulation of the genes tested, since the induction of the genes occurred in those bacteria inoculated only with bacteria- and cell-free media prepared from an another co-cultivation experiments. These results may indicate that CEICs and/or bacteria secrete components into the environment after physical contact event(s). The secreted component(s) may subsequently initiate the expression of the virulence-associated genes in other bacterium that had never been physically contacted with either CEICs or invaded/adhered bacteria. Thus, the up-regulation of these virulence-associated genes may not be bacteria-cell contact dependent, but the secretion of the active component(s) is bacteria-cell contact dependent.

## Conclusion

In summary, we have demonstrated that both *C. jejuni *strains of human and chicken origin can invade CEICs and stimulate CEICs to undergo an inflammatory response in a similar manner. The expression of virulence-associated genes of *C. jejuni *is increased during the course of invasion, and may play a role in the invasion process.

## Methods

### Bacteria

Two *C. jejuni *strains SC11, a Danish chicken isolate, and HM5040, a human isolate, were used in this study. These two strains were the most common serotype (Penner serotype 2) and *flaA *type (*flaA *type 1) among isolates from broilers and human cases in Denmark [39]. Both strains were taken from -80°C stock and streaked on modified CCDA (mCCDA) (blood-free agar base with cefoperazone [32 mg/liter] and amphotericin B [10 mg/liter]) (CM739 plus SR155; Oxoid, Basingstoke, UK) agar plates and incubated under microaerobic condition (10% CO2, 2–4% H2 and 86%–88% N2) at 42°C for 48 h. The bacteria were then sub-cultured on a new mCCDA plate for another 24 h prior to experimentation. The bacteria were harvested and well suspended in room temperature PBS (10 mM) to an OD_595 _= 1.0 (approximately 10^9 ^bacteria/ml). The bacteria were then diluted in pre-warmed Dulbecco's modified Eagle's growth medium (DMEM) (Invitrogen, UK) without antibiotics to the desired density for inoculation of cell cultures at a multiplicity of infection (MOI) of 100 bacteria per cell.

### Cell cultures

Primary chicken embryo intestinal cells (CEICs) were prepared from 19-day-old specific pathogen free (SPF) chicken embryos (Lohmann Tierzucht GmbH, Cuxhaven, Germany). Briefly, SPF chicken embryos were dissected, and intestines were separated and cut into small pieces in PBS prior to being digested with 0.25% trypsin-EDTA (Invitrogen, UK) for 3 times, 5 min for each. The cells were then collected by centrifugation at 500 × g for 10 min at room temperature (~25°C). The cells were seeded in 12-well plates at a density of 3.5 × 10^5 ^cells/ml (1 ml per well) in DMEM (Invitrogen) supplemented with 10% fetal bovine serum (FBS) (Invitrogen), 0.3% (w/v) tryptose phosphate broth (containing 0.13% pancreatic digest of casein, 0.07% proteose peptone No. 3, 0.02% dextrose, 0.05% sodium chloride, 0.026% disodium phosphate), and 0.02% penicillin and streptomycin each (Invitrogen). The cells were incubated in a humidified environment at 37°C with 5% CO2 throughout the experiment. At 24 h the medium was replaced with fresh growth medium, the cells were incubated for anther 72 h prior to bacterial inoculation experiments. The cells were washed three times with pre-warmed PBS, freshly prepared 300 μl of antibiotic-free medium containing the desired number of bacteria was added and co-cultivated with cells to various time points. All inoculations were carried out at a MOI of 100:1, unless stated otherwise. Controls consisted of mock infections using antibiotic-free medium alone or positive controls of lipopolysaccharide (LPS, 5 μg/ml) (Sigma, Schnelldorf, Germany).

To prepare bacteria- and cell-free cultured medium, cells were inoculated with the desired number of bacteria for 4 h (MOI = 100), the medium was collected, the bacteria was removed by centrifugation three times at 10,000 × g, 3 min for each, or filtration through a 0.22 μm filter. Three hundred μl of this medium was added to freshly harvested bacteria of ~1.7 × 10^7 ^and incubated for 4 h. As controls, the bacteria incubated with fresh and CEICs-cultured medium were included in parallel. The incubated bacteria were collected by centrifugation and subject to bacterial total RNA isolation as described below.

### Intracellular bacterial counts

The number of intracellular bacteria per eukaryotic cell culture was assessed by the gentamicin protection assay [8]. At 1, 2, 3, 4 and 24 h post inoculation (p.i.), the culture medium was removed, Hank's Buffered Salt Solution (HBSS, Invitrogen) containing gentamicin (200 μg/ml) was added, and incubated at 37°C, 5% CO2 for 2 h. The cells were then washed three times with pre-warmed PBS and lysed with 500 μl of 0.2% (v/v) Triton X-100 at room temperature for 15 min. The bacteria counts were numerated by plates counting and were present as a percentage of invaded bacteria to total inoculated bacteria.

### RNA extraction, reverse transcription, and PCR conditions

Total cellular and bacterial RNA was extracted using TRIZOL LS Reagent (Invitrogen) and re-purified using RNeasy Mini RNA isolation kit (Qiagen, Germany) according to the manufacturers's instructions. At 0, 1, 2, 3, 4 and 24 h p.i., both the medium of the inoculation and the suspended bacteria were collected by centrifugation at 10,000 × g for 3 min. The bacteria pellets and cells were lysed in 300 μl of TRIZOL LS reagent and proceed to RNA isolation. RNA was eluted into 50 μl of RNase-free water and treated with 0.1 U/ml Dnase I Amplification Grade (Invitrogen) according to the manufacturer's protocol. The treated RNA was further tested for DNA contamination by PCR using the primer pairs of β-actin and 16S rRNA (Table 1). DNA-free RNAs were transcribed to complementary DNA (cDNA) using the iScript™ cDNA Synthesis Kit (Bio-Rad, USA) with pre-blended RNase inhibitor, oligo (dT) and random hexamer primers, according to the manufacturer's instructions. Quantitative real time PCR (qPCR) was performed in the Mx3005P thermocycler (Strategene, Denmark) using primers listed in Table 1. PCR mixtures (25 μl) contained 0.625 units of Taq DNA Polymerase (Promega, Denmark), 5 mM MgCl2, 200 μM dNTPs, 400 nM of each primer, 10 nM fluorescein (Bio-Rad), 50000 × diluted SYBR green (Invitrogen), and 2 μl of diluted cDNA. The thermal cycling conditions included an initial heat-denaturing step at 94°C for 3 min; 45 cycles of 94°C for 15 s, optimal annealing temperature for appropriate primer pair (Table 1) for 20 s and 72°C for 20 s; followed by an elongation step at 72°C for 3 min. A single fluorescence measurement was taken after each thermo cycle. The specificity of amplifications was checked by melting curve analyses and 2% agarose gel. All primers gave specific melting curves and electrophoresis bands as expected (data not shown). The efficiency of the amplification for each primer pair was assessed by a slope of the equation of a standard curve generated from the serial dilutions of the pooled samples. A slope of -3.32 indicates the PCR reaction has 100% efficiency [40]. The slopes of all primer pairs are close to each other, from -3.35 to -3.12 (Table 1), suggesting a close efficiency of the amplification between reference and target genes in qPCR. The close efficiency of amplification between target and reference validates the quantification of genes by either the standard curve or the 2^-ΔΔCt ^methods in further experiments.

**Table 1:** Genes and primers used in this study.

Genes (Species)	Primer sequences (5'-3')	Annealing temperatures (°C)	Amplicons (bp)	Standard curves	Correlation coefficient (r^2^)	GenBank access no.
β-actin (*Gallus gallus*/chicken)	GAGAAATTGTGCGTGACATCA CCTGAACCTCTCATTGCCA	63	152	y=-3.312x+19.3	0.997	L08165
IL-1β(*Gallus gallus*/chicken)	CTGGGCATCAAGGGCTACA GGCTGTCCAGGCGGTAGA	62	142	y=-3.359x+25.3	0.990	AJ245728
IL-6 (*Gallus gallus*/chicken)	GTTCGCCTTTCAGACCTAC ACCACTTCATCGGGATTTA	60	138	y=-3.288x+23.1	0.985	AJ309540
CXCLi1 (K60) (*Gallus gallus*/chicken)	CAAGCACGTTCAGCGATT ATTCTTGCAGTGAGGTCCG	57	117	y=-3.246x+24.79	0.994	AF277660
CXCLi2 (CAF/IL-8) (*Gallus gallus*/chicken)	TTGGAAGCCACTTCAGTCAGAC GGAGCAGGAGGAATTACCAGTT	60	120	y=-3.234x+22.5	0.993	AJ009800
TGF-β4 (*Gallus gallus*/chicken)	AGGATCTGCAGTGGAAGTGGAT CCCCGGGTTGTGTTGGT	61	137	y=-3.145x+23.54	0.990	M31160
iNOS (*Gallus gallus*/chicken)	CAGCTGATTGGGTGTGGAT TTTCTTTGGCCTACGGGTC	58	158	y=-3.245x+25.8	0.944	U46504
16S (*C. jejuni*) rRNA (*C. jejuni*)	AACCTTACCTGGGCTTGATA CTTAACCCAACATCTCACGA	52	122	y=-3.149x+15.7	0.998	NC_002163
*ciaB *(*C. jejuni*)	ATATTTGCTAGCAGCGAAGAG GATGTCCCACTTGTAAAGGTG	51	157	y=-3.357x+23.7	0.996	NC_002163
*dnaJ *(*C. jejuni*)	AGTGTCGAGCTTAATATCCC GGCGATGATCTTAACATACA	51	117	y=-3.166x+26.3	0.996	NC_002163
*racR *(*C. jejuni*)	CTTAAGCGATAAAGTTGTGG CTTTTTGTGCGACGAAT	51	114	y=-3.122x+23.7	0.996	NC_002163

### Quantification of cytokines and bacterial virulence-associated genes

The transcripts of cytokine interleukin 1β (IL-1β), IL-6, CXCLi1, CXCLi2, tumor growth factor β4 (TGF-β4) and the inducible nitric oxide synthese (iNOS) were measured in inoculated and mock-inoculated cells using qPCR relative to β-actin. The transcripts of bacterial virulence-associated genes *ciaB*, *dnaJ *and *racR *in invaded/adhered, suspended and those bacteria treated with various media were quantitatively determined by qPCR relative to 16S rRNA, by use of the 2^-ΔΔCt ^method previously described [41]. PCR was performed in duplicate including no-template controls.

### Measurement of NO production

Nitric oxide production was determined by measuring nitrite in cell culture media using the Griess Reagent System (Promega) assay according to the manufacture's instruction. The absorbance was read at 550 nm, using a Multiskan EX microtiter plate reader (Thermo Labsystem, Multiskan EX, Denmark).

### Statistical analyses

The values were expressed as the average ± standard deviation (SD). The data were analyzed for statistical significance using one-way ANOVA (ANalysis Of VAriance, Microsoft Excel). A *p*-value ≤ 0.05 was considered to be statistically significant.

## Authors' contributions

YPL participated in the development of study design, performed the experiments, collected and interpreted the data, and wrote the manuscripts. HI and MM contributed to the editing of the manuscript and discussions. DDB participated in the development of study design and discussions, interpreted the data and editing the manuscript. All authors read and approved the final manuscript.
